# Updated and New Perspectives on Diagnosis, Prognosis, and Therapy of Malignant Pheochromocytoma/Paraganglioma

**DOI:** 10.1155/2012/872713

**Published:** 2012-07-17

**Authors:** Gabriele Parenti, Benedetta Zampetti, Elena Rapizzi, Tonino Ercolino, Valentino Giachè, Massimo Mannelli

**Affiliations:** ^1^Endocrinology Unit, Azienda Ospedaliero-Universitaria Careggi, Largo Brambilla 3, 50134 Florence, Italy; ^2^Department of Clinical Pathophysiology, University of Florence, Viale Pieraccini 6, 50139 Florence, Italy; ^3^Istituto Toscano Tumori, Via Taddeo Alderotti 26N, 50139 Florence, Italy

## Abstract

Malignant pheochromocytomas/paragangliomas are rare tumors with a poor prognosis. Malignancy is diagnosed by the development of metastases as evidenced by recurrences in sites normally devoid of chromaffin tissue. Histopathological, biochemical, molecular and genetic markers offer only information on potential risk of metastatic spread. Large size, extraadrenal location, dopamine secretion, *SDHB* mutations, a PASS score higher than 6, a high Ki-67 index are indexes for potential malignancy. Metastases can be present at first diagnosis or occur years after primary surgery. Measurement of plasma and/or urinary metanephrine, normetanephrine and metoxytyramine are recommended for biochemical diagnosis. Anatomical and functional imaging using different radionuclides are necessary for localization of tumor and metastases. Metastatic pheochromocytomas/paragangliomas is incurable. When possible, surgical debulking of primary tumor is recommended as well as surgical or radiosurgical removal of metastases. I-131-MIBG radiotherapy is the treatment of choice although results are limited. Chemotherapy is reserved to more advanced disease stages. Recent genetic studies have highlighted the main pathways involved in pheochromocytomas/paragangliomas pathogenesis thus suggesting the use of targeted therapy which, nevertheless, has still to be validated. Large cooperative studies on tissue specimens and clinical trials in large cohorts of patients are necessary to achieve better therapeutic tools and improve patient prognosis.

## 1. Introduction

Paragangliomas (PGLs) are rare neuroendocrine tumors that arise in sympathetic and parasympathetic paraganglia and derive from neural crest cells. Approximately 80–85% of these tumour arise from the adrenal medulla and are named pheochromocytomas (PCCs), whereas 15–20% are located in extra-adrenal chromaffin tissue and are referred to as secreting paragangliomas (sPGLs). The latter term is also used to describe tumors derived from parasympathetic tissue in the head and neck (HNPGLs).

PCCs and abdominal sPGLs are usually catecholamine-producing tumours, whereas most of the HNPGLs are nonfunctioning [1].

The majority of PGLs are sporadic, but recent data have demonstrated a high prevalence of hereditary forms (approximately 35%) [2]. Sporadic PGLs are usually diagnosed in patients older than 40–50 years, whereas hereditary forms are diagnosed in younger patients.

Malignancy is defined by presence of metastases, tumor spread in sites where chromaffin tissue is normally absent such as lymph nodes, liver, lungs, and bones. Malignant PGLs are extremely rare. An estimated incidence in USA in 2002 was 93 cases per 400 million persons [3].

Nearly 10% and 20% of PCCs and abdominal sPGLs, respectively, are malignant [4], whereas HNPGLs are usually benign [5].

## 2. Clinical Feature

The high variability in clinical presentation of PGLs is well known [6]. It depends on the variability in the biology of these tumours which can express different catecholamine biosynthetic enzymes, secrete different vasoactive peptide (i.e., neuropeptide Y, adrenomedullin, or atrial natriuretic peptide) [7], present different symptoms related to tumour mass or present symptoms related to other organs involvement in syndromic forms.

Hypertension is the most common feature of PCCs and sPGL: it can be continuous, intermittent, and often paroxysmal in nature. Hypertensive crises are frequently associated with the classic triad of severe headache, palpitations, and diaphoresis. Other signs or symptoms such as dyspnoea, weakness, arrhythmias, visual disturbances and metabolic effect such as glucose intolerance and weight loss are reported [1]. The cardiovascular complications (sudden death, myocardial infarction, hearth failure, and cerebrovascular accidents) represent the most frequent causes of morbidity and mortality in these patients.

HNPGLs are usually clinically silent, but they can determine manifestations related to mass effect or infiltration of the adjacent structures. In such situations the presence of a palpable neck mass as well as pain, dysphagia, tinnitus, or cranial nerve palsies has been reported [8].

In addition to the abovementioned symptoms and signs, malignant PGLs may also present “systemic” symptoms (anorexia, fatigue, and weight loss) or clinical manifestations related to the metastatic disease such as pain in bones affected by metastatic spread. Malignant PGLs, being less-differentiated tumours with a less differentiated biosynthetic pathway, generally secrete noradrenaline and/or dopamine causing even milder cardiovascular symptoms and a subclinical picture [9]. Metastatic spread may occur at presentation or even after many years from primary surgery.

## 3. Diagnosis: Biochemistry

The recommended screening test for initial assessment of PGLs is the measurement of plasma free-metanephrines or urine-deconjugated differential metanephrines [10]. In fact in comparison to plasma or urine catecohlamines and vanilmandelic acid, metanephrines show higher sensitivity, ranging around 98-99% [11, 12]. This is mainly related to their longer half-life and to their continuous production by the tumour where catecholamines are converted to metanephrines by the high methyltransferase activity of the chromaffin tissue [13].

The biochemical phenotype does not permit to differentiate malignant from benign PGLs.

PGLs exhibit different biochemical properties as PCCs mainly produce adrenaline, while sPGLs secrete noradrenaline. Malignant PCCs secrete predominantly noradrenaline [14], but due to an even less-differentiated catecholamine biosynthetic pathway, they may often produce mainly or exclusively dopamine [15]. Therefore, the presence of large predominantly noradrenaline-producing PGLs and increased levels of plasma dopamine or its metabolite methoxytyramine may suggest malignancy [11, 16].

Plasma chromogranin A (CgA), a protein stored and co-secreted with catecholamines, is often increased in functioning and nonfunctioning PGLs [17]. CgA shows a sensitivity of 83–89% for identifying PGLs, but it often shows false positive results because of liver or kidney failure or proton pump inhibitor therapy [18].

Malignancy is generally associated to very high plasma levels of CgA [11].

High plasma levels of neuron-specific enolase are sometimes found in patients with malignant PGLs [19, 20], while overexpression of secretogranin II and prohormone convertases I and II suggests a benign lesion [21].

## 4. Diagnosis: Anatomical and Functional Imaging

Anatomical imaging such as computed tomography (CT) or magnetic resonance imaging (MRI) is useful as the first radiological approach in patients with PGLs. CT shows a sensitivity of 77–98% and a specificity of 29–92% in the localization of adrenal or extra-adrenal tumors. A slightly better accuracy (sensitivity 90–100% and specificity 50–100%) has been reported for MRI, especially for the detection of extra-adrenal disease [22].

PGLs are highly vascular tumors with a high intracellular water content and frequent intratumoral cystic lesions, which show a typical, but not diagnostic, high signal on T2-weighted imaging, and strong enhancement after contrast-agent administration. Nevertheless, in large tumors with haemorrhagic and/or necrotic areas (features often detected in malignant lesions), the signal intensity on T2-weighted images may be low [23].

Ultrasound imaging is of limited diagnostic yield but can be useful for the detection of HNPGLs [22, 24].

After “anatomical” imaging, a “functional” imaging is generally recommended. ^131^I or ^123^I-metaiodobenzylguanidine (MIBG) scintigraphy has been used extensively as a first-line nuclear medicine technique in evaluation of patients with PGLs. MIBG has chemical similarities to norepinephrine and is concentrated in chromaffin tissue, via the human norepinephrine transporter (hNET), that is expressed in most of chromaffin cells and it is normally responsible for catecholamines uptake [25].

^123^I-MIBG is superior to ^131^I-MIBG in terms of physical properties, quality of images, and sensitivity. ^123^I-MIBG scanning shows a sensitivity of 83–100% and a specificity of 95–100% [22]. The possibility to perform a whole-body study, may permit a better evaluation of extra-adrenal localization of the disease as well as of multiple tumors and/or metastatic sites [25]. The sensitivity of this technique in malignant PGLs may be lower as evidenced in situation highly associated with malignancy as in *SDHB *mutation carriers (see later) or patients with dopamine-secreting tumours which usually do not uptake MIBG [26].

In patients with negative MIBG scintigraphy, other tracers may be used. The expression of somatostatin receptors (SSTRs), especially SSTR 2, 3, and 5 on chromaffin cells, represents the rationale for the use of radiolabelled somatostatin analogues in localization of these tumors. Indium-11-DTPA-octreotide (^111^In-penteotride) is the tracer most commonly used; it is of limited value in benign PCCs, but it may be useful in detecting extra-adrenal disease as well as MIBG-negative metastases. In fact a sensitivity near to 90% has been reported for localizing sPGLs, HNPGLs, or malignant PCCs [22, 27].

Somatostatin analogues labelled with gallium-68 can be used in PET imaging; ^68^Ga-DOTATOC (DOTA^0^-D-Phe^1^-Tyr^3^-octreotide) has shown a better sensitivity than ^111^In-penteotride in the detection of neuroendocrine tumors especially in detecting small lesions or neoplasms bearing only a low density of SSTR. Moreover, it permits a better identification of metastases located in the lung or in the skeleton. In relation to PGLs, this tracer seems superior to ^18^F-labelled fluoro-deoxy-glucose (^18^F-FDG) in detecting malignant PCCs and sPGLs [28–30].

Radiolabelled dopamine (DA) or dihydroxypheylalanine (DOPA) which are transported in to chromaffin cells by hNET, may be used as tracers in positron emission tomography (PET) imaging. PET with 6-[^18^F]-fluoro-DA can detect metastatic PCCs with better sensitivity than ^131^I-MIBG [31], whereas PET with 6-[^18^F]-fluoroDOPA is superior in imaging sPGLs and HNPGLs [32]. However, as for MIBG, these tracers show a relative low sensitivity (70–88%) in PGLs associated with *SDHB* gene mutations. In such conditions PET with ^18^F-FDG shows a higher sensitivity (97–100%) [33]; this PET scanning is useful in identifying glucose-avid metastatic lesions, particularly if they are MIBG-negative [34].

Finally PET imaging with ^11^C-hydroxyephedrine has provided high sensitivity and specificity (92 and 100%, resp.) in the detection of PGLs, but the small number of patients studied makes not possible to draw conclusions on its utility [35].

## 5. Diagnosis: Histopathologic and Molecular Markers

Despite the increasing availability of molecular diagnostic and prognostic markers, it remains difficult to predict, on the basis of histological findings, whether an apparently benign PGL will develop in a malignant tumor. From a prognostic point of view, only relative risk factors can be taken into account. In general PGLs larger than 5 cm with necrotic areas as well as extra-adrenal tumors carry a higher risk of malignancy than neoplasms that are small or located in the adrenal. Several scoring systems considering invasion, histologic growth patterns, cytologic features, or mitotic activity have been proposed to calculate the risk of malignancy [36–38]. One of the most utilized score is “Pheochromocytoma of the Adrenal gland Scales Score (PASS),” proposed by Thompson on 2002.  Table 1 reports the items and their values which are necessary to calculate the PASS. A PASS score ≥4 was at first considered suggestive for a biological aggressive behaviour, but a later study revealed that all malignant PCCs had a PASS >6 [39]. On the basis of these results a PASS score <4 or >6 suggest benign and malignant lesions respectively, whereas a value between 4 and 6 suggests an intermediate risk. In any event, as none of the available scores predicts malignant development unequivocally, after the removal of an isolated primary PGL, a followup of the patient is recommended in order to reveal early disease recurrence. Between histological features, high cellularity and particularly the presence of tumor necrosis are considered potential indicators of malignancy.

Further information can derive from the evaluation of specific molecular markers. Several malignancy tissue markers such as cyclooxygenase-2, secretogranin II-derived peptide, *N*-cadherin, vascular endothelial growth factor (VEGF), endothelin receptor type A (ETA), and type B (ETB) and telomerase have been identified. In particular telomerase, which is a ribonucleoprotein complex that includes the telomerase RNA component, the telomerase-associated protein (TP1), the telomerase catalytic subunit (hTERT), and the heat shock protein 90 (HSP90) seem to be closely related to the malignant potential of PGLs. In fact an upregulation of hTERT, HSP90, and telomerase activity has been evidenced in malignant cells of PCCs [40].

The Ki-67 nuclear antigen represents another potential molecular marker which has been associated with more aggressive cancers. A Ki-67 index >3% is considered a useful parameter predicting malignant potential [41].

Another promising marker predicting metastatic potential seems the transcription factor SNAIL. Positive immunostaining has been found significantly higher in metastatic than benign PGLs [42, 43].

Novel biomarkers are recently being identified by micro-RNA expression profiling studies. Micro-RNA is small single-strand (~22 bp), nonprotein coding RNA fragments, which are able to negatively regulate protein expression by either cleavage or translational repression of mRNA [44]. Recently, Meyer-Rochow, and colleagues [45] investigated 12 malignant, 12 benign tumors, and 5 healthy adrenal medulla samples. They found that miR-483-5p was overexpressed, while miR15a and miR-16, which are involved in proliferation and apoptosis, were downregulated in malignant compared to benign tumors. MicroRNA expression is tissue specific, and it has been demonstrated to be altered in several other human tumors, For these reasons, they can be of great relevance for the establishment of malignancy, but further investigations in larger cohorts of patients are necessary to confirm these encouraging results.

## 6. Diagnosis: Genetic Aspects

Until 2000, only 10% of PGLs were considered of genetic origin and linked to hereditary syndromes: von Hippel Lindau disease (VHL), multiple endocrine neoplasia type 2 (MEN2) and neurofibromatosis type 1 (NF1), due respectively to a germ line mutation in tumor-suppressor gene *VHL* [46, 47], protooncogene *RET* [48–52] and tumor-suppressor gene *NF1 *[53].

In the last years it has been demonstrated that about 30% of the apparently sporadic PGLs are due to a germ-line mutation in one of the susceptibility genes [54]. This group of genes includes those encoding the four subunits (A, B, C, and D) of the succinate dehydrogenase (SDH) [55–58], the recently identified gene *SDHAF2*, which is responsible for the flavination of the SDHA subunit [59], and the very recently discovered *TMEM127* [60] and *MAX* [61], both mainly related to bilateral PCCs. Germ line mutations in *SDHA, SDHB, SDHC*, *SDHD*, and *SDHAF2 *genes are responsible for the occurrence of syndromes named PGL5, PGL4, PGL3, PGL1, and PGL2, respectively; to note, *SDHB*-mutations are generally associated with higher morbidity and mortality than mutations in the other SDHx genes [62]. A recent meta-analisis of some studies involving *SDHB* mutated patients has highlighted that 31% of their tumors were malignant [3].

Overall, to date, 10 susceptibility PGLs genes have been identified, so that the initial 10% of cases classified as genetically determined has increased to 30%. Nevertheless, the number of the susceptibility genes is likely to increase. In fact, many young PGL patients, where the mutation frequency is higher, are still classified as sporadic, and some PGLs patients with a positive family history do not show any mutation in the so far known susceptibility genes.

Extensive genetic screening in PGLs has highlighted the correlation between genotype and phenotype thus facilitating a genetic testing algorithm based on clinical features as a guide for a more quick and cost-effective genetic screening (Table 2) [63].

Genetic analysis has also permitted to predict the malignancy risk which is higher for SDHB mutation carriers.

Furthermore, by studying tumor transcription profile, sporadic as well as hereditary PGLs have been divided in two main clusters linked to two different signalling pathways [64]: the first cluster contains all *VHL*- and *SDHx*-mutated tumors and is associated with angiogenesis, hipoxia, and reduced oxidative response [65], while the second cluster contains all *RET*- and *NF1*-mutated tumors and is associated with abnormal activation of kinase-signaling pathways, such as RAS/RAF/MAPK and PI3K/AKT/mTOR [66–69]; also *TMEM127* [60] and *MAX* [61] mutated tumors have been associated to the activation of mTOR-signaling pathway. These data have increased overall knowledge on molecular defects in PGLs and could be used for development of new effective molecular-targeted therapies.

## 7. Therapy: Surgery

The main goal of surgical treatment is represented by the removal of primary tumor and, when possible, the resection of local and distant metastases. The overall 5-year survival rate of patients with malignant PGLs varies between 34% and 60%. The survival rate may depend upon sites of metastatic lesions. In fact, patients with liver or lung metastases tend to have a worse prognosis (<5 years) than patients with isolated bone lesions [2].

The preoperative management with alpha blockade and fluid administration, essential in order to avoid surgical (i.e., hypertensive crisis arrhythmias) and/or postsurgical complications (i.e., hypotension), has to be performed in all patients [1].

Laparoscopic removal of intra-adrenal and extra-adrenal PGLs is the preferred surgical technique, but, in case of large tumors with a high risk of malignancy, a transabdominal approach should be considered. In such circumstances total adrenalectomy with resection of locoregional lymph nodes or complete excision of PGLs together with the removal of distant metastases is recommended [70]. In case of malignant disease surgery alone is seldom curative, but surgical debulking of the tumors is regarded as a mainstay of palliative therapy. In fact, it permits to reduce local or systemic symptoms related to catecholamine secretion, it improves response to other therapeutic approaches and it may prevent further diffusion of the tumors. Pre-operative injection of ^123^I-MIBG and intraoperative application of a **γ**-probe may permit the localization of lesions that are not evidenced by other imaging techniques [71].

In the presence of liver metastases arterial embolisation or chemoembolisation may provide transient response, but in such circumstances radiofrequency ablation has become the preferred choice [72]. In the next future, the rapidly evolving stereotaxic radiotherapic techniques will probably represent valid alternative tools for metastasis removal.

## 8. Therapy: Radiometabolic Treatment and External Radiotherapy

Radionuclide treatment may be considered in patients with metastatic disease and no resectable lesions. It can be performed using beta-emitting isotopes coupled with MIBG or somatostatine analogue.

^131^I-MIBG was used in the treatment of malignant PCC for the first time in 1984 [73]. Patients are selected by the evidence of significant radioisotope uptake on diagnostic scintigraphy with ^123^I-MIBG or ^131^I-MIBG (>1% uptake of the injected dose). Single or fractionated doses as well as variable dosage (200–1400 mCi) have been proposed [74, 75]. About 60% of metastatic sites are ^131^I-MIBG avid [76]. In general better responses are seen in patients with limited disease and in patients with soft-tissue metastases than in patients with bone metastases [77]. This treatment is well tolerated, and the main side effects are represented by transient leucopenia and thrombocytopenia, whereas severe bone marrow toxicity (associated with high-dose regimen) is rarely seen.

However treatment with ^131^I-MIBG is not curative in most patients; therefore, other forms of therapy need to be considered.

The presence of SSTR in PGLs has allowed treatment with radiolabelled somatostatin analogues. The most commonly used are Yttrium-90-DOTATOC (^90^Y-DOTA-TOC) and Lutetium-177-DOTA^0^-Tyr^3^-octreotate (^177^Lu-DOTA-TATE) [75, 78, 79]. As for ^131^I-MIBG, patients are selected by the demonstration of high tumor uptake at scintigraphy. The latter is usually performed with ^111^In-pentetreotide, but it seems that PET using ^68^Ga-DOTA-TOC provides higher accuracy in selecting patients [34]. This kind of therapy has a low toxicity, mainly leucopenia and thrombocytopenia, and it can be effective in order to reduce hormone secretion and determine tumor shrinkage. Therefore, even if its efficacy seems lower in PGLs than in gastroenteropancreatic neuroendocrine tumors, it represents an alternative option for the treatment of surgically incurable PGLs [80]. In the future, the development of new somatostatine analogues with higher affinity for the different SSTR subtypes will provide a further possibility in the treatment of these neoplasms.

Finally a combined treatment with radiolabelled MIBG and radiolabelled somatostatin analogues might have a synergistic effect, and therefore it might be considered. Moreover, the combination treatment could permit the use of lower doses of both radionuclides, limiting side effects, particularly bone marrow toxicity.

External radiotherapy may be considered for treatment of inoperable PGLs and especially for palliation of painful bone metastases. During this procedure the patients need to be monitored because the radio-induced inflammation of the lesion can induce massive catecholamine secretion, thus inducing hypertensive crises [81].

## 9. Therapy: Antineoplastic Agents

The aim of chemotherapy is tumor size reduction and control of symptoms due to catecholamine secretion; it is usually reserved to patients with local advanced and/or metastatic disease, with unresectable lesions, resistant to treatment with radionuclide therapy [82]. Up to now, the most used and effective chemotherapy regimen is a combination of cyclophosphamide, vincristine, and daecarbazine (CVD), chosen for its use in treating another neuroendocrine tumor, neuroblastoma. It has been used for the first time in 1980s in a trial including 14 malignant PCCs cases [83], updated recently by NIH [84] in a 22-year followup, with demonstration of a tumor regression and symptom relief in up to 50% of patients treated and no significant change of survival. Once CVD is stopped, PCCs often recur, becoming unresponsive to the same treatment. For these reasons, CVD may have a role as a neoadjuvant therapy in few cases, to make tumors surgically resectable and to control symptoms. CVD plus anthracyclines has been tested in one case with quite a good result [85]. Other chemotherapic regimens have been tested in other trials, but currently none has demonstrated effectiveness in malignant PCCs treatment [86].

## 10. Therapy: Targeted Approach

Up to now, treatment options for malignant PGLs are limited to chemotherapy and radionuclide therapy. These often provide symptomatic and biochemical control but are less effective in causing survival increase. Understanding specific molecular pathways alteration responsible for malignant PGLs development might hopefully in the future lead to multiple molecular-targeted therapy for a successful treatment. Effectiveness of these therapies is due to a cytostatic effect, as they interfere with specific molecular targets found along the oncogenic signaling pathways responsible for carcinogenesis and tumor growth. As stated above, both benign and malignant PGLs gene mutations are part of two distinct molecular pathways leading to tumorigenesis: cluster 1 includes mutations of *VHL*, *SDHB*, and *SDHD* and is associated to pseudohypoxia and aberrant VEGF signaling, leading to abnormal hypoxia inducible factor (HIF) activation and overexpression of angiogenic factors, while cluster 2 includes mutations of *RET, NF1*, *TMEM127*, and *MAX* and is associated with abnormal activation of kinase-signaling pathways such as PI3kinase/AKT, RAS/RAF/ERK, and mTOR1/p70s6K, leading to abnormal cell growth and lack of apoptosis capacity. In addition, malignant PCCs seem to overexpress HSP90, a molecular chaperone that assists in folding proteins and stabilizes various oncoproteins that play a role in malignant phenotype [87, 88].

Thus, HIF1a inihbitors are molecular targeted drugs interfering with HIF hypoxia-driven transcription pathway, decreasing HIF activity directly, PX-478 (S-2-amino-3-[4′-N,N,-bis (2-chloroethyl)amino]-phenyl propionic acid N-oxided ihydrochloride), and indirectly, PX-12 (1-methylpropyl 2-imidazolyl disulfide). These agents have shown marked antitumoral activity in human tumor xenografts in mice and seem to be promising also for malignant PGLs, but conclusive data are missing [89–91].

The mTOR inhibitor everolimus (RAD001) in combination with octreotide has been evaluated for low- and intermediate-grade neuroendocrine tumors [92], with good results. The efficacy of everolimus has been evaluated also in malignant PGLs, but all patients experienced disease progression [4, 93]. Maybe the low efficacy is due to a compensatory PI3K/AKT and ERK activation in response to mTOR inhibition, so a specific novel dual PI3k/mTOR inhibitor (NVP-BEZ235) might offer a novel therapeutic approach [94]. Further studies on the PI3K/AKT/mTOR pathway have to be conducted to find a more specific molecular target in its signalling.

Due to overexpression of HSP90 in malignant PCCs [40, 95], inhibition of its pathway could represent a future therapeutic challenge for the treatment of malignant PCCs, but at present current specific drug trials are missing.

Several studies have demonstrated overexpression in malignant PCCs of angiogenic molecules, such as VEGF, its receptor, angiopoietin-2, and the endothelin receptors ETA and ETB [96–100], leading a strong evidence that targeting this pathway with antiangiogenic therapies could represent a new promising treatment option. Accordingly, sunitinib, a receptor tyrosine kinase inhibitor acting on several targets (VEGF, PDGF, and c-KIT), with strong antiangiogenic and antitumor activity, has been used in the treatment of malignant PCCs, with promising results [101–104].

Imatinib, another tyrosine kinase inhibitor already used for hematologic and gastrointestinal stromal tumors, has not been found effective for malignant PCCs treatment [105].

Thalidomide, by targeting VEGF and basic fibroblast growth factor, is an antiangiogenic agent evaluated for treatment of metastatic renal cell cancer, multiple myeloma and nonsmall cell lung cancer [106, 107]. It has been used in combination with Temozolomide in neuroendocrine tumors [108] obtaining an objective biochemical response rate in about 40% and a radiologic response rate in 33% of malignant PCCs, but lymphopenia occurred in about 70% of treated patients.

Activators of prolyl hydroxylase (PHD) (such as ERBB2 inhibitors) are now on evaluation as promising antineoplastic therapies. These molecules decrease the expression levels of some angiogenic factors, such as VEGF, acting on HIF pathway, by activating the PHD, thus increasing HIF hydroxylation, and promoting its degradation [109, 110].

Treatment of malignant PGLs is up to now basically palliative. Molecular targeted therapies are promising strategies, but, due to the complexity of these tumors pathogenesis, further studies on tumor biology, discovery of novel targeted drugs, and new trials are needed to achieve more effective treatments.

## 11. Conclusions

Malignant PGLs, as defined by the presence of metastases, are very rare and aggressive tumors. Their study is made difficult by their rarity, and the consequent limited number of patients included in the series, by their biological variability, by their variable genetic background, and by the lack of specific and sensitive histopathological or biological markers proving malignancy. Therefore, as benign tumors are diagnosed by the lack of metastases, and as metastatic spread can occur also several years after surgical removal of primary tumor, studies comparing benign and malignant PGLs need a long clinical followup of patients.

Large collaborative international studies, as those presently conducted on behalf of the European Network for the Study of Adrenal Tumors (ENS@T), are needed to improve our knowledge on the pathogenesis of these malignant tumors and to achieve a satisfactory medical treatment for affected patients.

## Figures and Tables

**Table 1:** Pheochromocytoma of the adrenal gland scoring scale (PASS) [38].

Items	Value
Nuclear hyperchromasia	1
Profound nuclear pleomorphism	1
Capsular invasion	1
Vascular invasion	1
Extension into adipose tissue	2
Atypical mitotic figures	2
Greater than 3 of 10 mitotic figures high-power field	2
Tumor cell spindling	2
Cellular monotony	2
High cellularity	2
Central or confluent tumor necrosis	2
Large nests or diffuse growth (>10% of tumor volume)	2

Total	20

**Table 2:** Correlations between gene mutations and clinical phenotype.

Syndrome	Gene	PCCs (%)	Sympathetic PGL	Parasympathetic PGL	Bilateral/multifocal neoplasia	Malignancy (%)
MEN 2A	*RET*	~50	Very rare	Extremely rare	+	<3
MEN 2B	*RET*	~50	Very rare	Extremely rare	+	<3
VHL	*VHL*	10–20	+	Rare	+	5
NF1	*NF1*	5	−	−	−	11
PGL1	*SDHD*	+	+	+	+	~5
PGL2	*SDHAF2*	−	−	+	+	Not known
PGL3	*SDHC*	−	Rare	+	−	Not known
PGL4	*SDHB*	Rare	+	Rare	+	~40
PGL5	*SDHA*	−	+	−	Not known	Not known
TMEM127 mutation carriers	*TMEM127*	100	−	−	+	~5
MAX mutation carriers	*MAX*	100	+	Extremely rare	+	~10

PCCs: pheochromocytomas; PGL: paragangliomas; +: present; −: absent.
